# Severe Gliosis Over a Closed Macular Hole Following Anterior Lens Capsular Flap Closure

**DOI:** 10.7759/cureus.54190

**Published:** 2024-02-14

**Authors:** Shreyas Temkar, Goutham Rajasekar, Jagadeeshwari Jayaseelan, Amit K Deb, Hemanth Ramachandar

**Affiliations:** 1 Ophthalmology, Jawaharlal Institute of Postgraduate Medical Education & Research, Puducherry, IND

**Keywords:** ilm peeling, pars plana vitrectomy, non-closed macular hole, anterior lens capsule, full-thickness macular hole

## Abstract

Various management strategies, including the use of autologous and allogenic materials, are described for the management of persistent macular holes. An anterior lens capsular flap can be used, especially when cataract surgery is also planned, for a persistent full-thickness macular hole. We report a case of a gentleman in his 60s who underwent anterior lens capsular flap closure for a persistent macular hole. There was an improvement in visual acuity. However, he developed severe gliosis over the closed hole in the postoperative period. This could be due to the proliferation of residual epithelial cells in the lens capsule, micro damage to the retina, or an exaggerated inflammatory response to a foreign tissue placed over the retinal surface.

## Introduction

Pars plana vitrectomy (PPV) with internal limiting membrane (ILM) peeling is the surgery of choice for the treatment of full-thickness macular holes (FTMH) [1]. Hole closure is achieved in 88-100% of cases [2,3]. Persistent macular holes following surgery can occur when the diameter of the hole is large (usually more than 400 µm) or when the macular hole index (MHI) is less than 0.5. Various surgical options are available for the treatment of such persistent macular holes. These include extension of the ILM peel, creation of a new ILM flap, pedunculated ILM flap, lens capsular flap, retinal graft, blood and platelet concentrate, or use of allogeneic tissues like human amniotic membrane. A few retinal manipulative procedures are also described like massage of hole edges, induction of macular detachment, and creation of retinal incisions [4-6]. The use of anterior or posterior lens capsules has been well described for the treatment of persistent macular holes [7-9]. This is effective in cases where extensive ILM peeling has already been done and no more ILM is available to be used as a graft. The use of a lens capsular flap usually achieves good success with no major complications [7-11]. We describe an unusual presentation of severe macular gliosis after performing anterior lens capsular flap transplantation for a case of persistent macular hole.

## Case presentation

**Figure 1:**
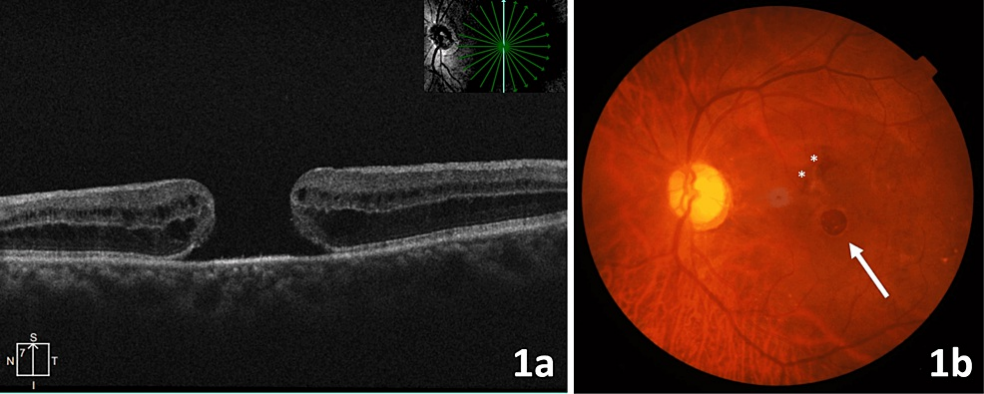
Left eye OCT at presentation showing a large full-thickness macular hole with a minimum linear diameter of 870 µm (A). Left eye fundus photo three weeks after initial macular hole surgery showing persistent macular hole (white arrow). Few residual petechial retinal hemorrhages from iatrogenic injury during ILM peeling are seen superior to the fovea, marked with white asterisks (B). OCT - optical coherence tomography; ILM - internal limiting membrane.

A gentleman in his 60s presented with a history of gradual onset, painless, progressive diminution of vision in the left eye for the past six months. His best corrected visual acuity was 20/20 in the right eye and 20/400 in the left eye. On evaluation, he was diagnosed with an idiopathic FTMH in his left eye. Optical coherence tomography (OCT) of the macula of the left eye confirmed the presence of FTMH with a minimum linear diameter of 870 µm and an MHI of 0.27 (Figure 1A). Since the hole was large with a lesser MHI, ILM peeling with an inverted ILM flap was done in addition to PPV and sulfur hexafluoride (SF6) gas tamponade. Despite strict prone positioning, there was no improvement in vision with the persistence of the macular hole (Figure 1B). The patient also developed a fine central posterior subcapsular cataract in the postoperative period.

**Figure 2:**
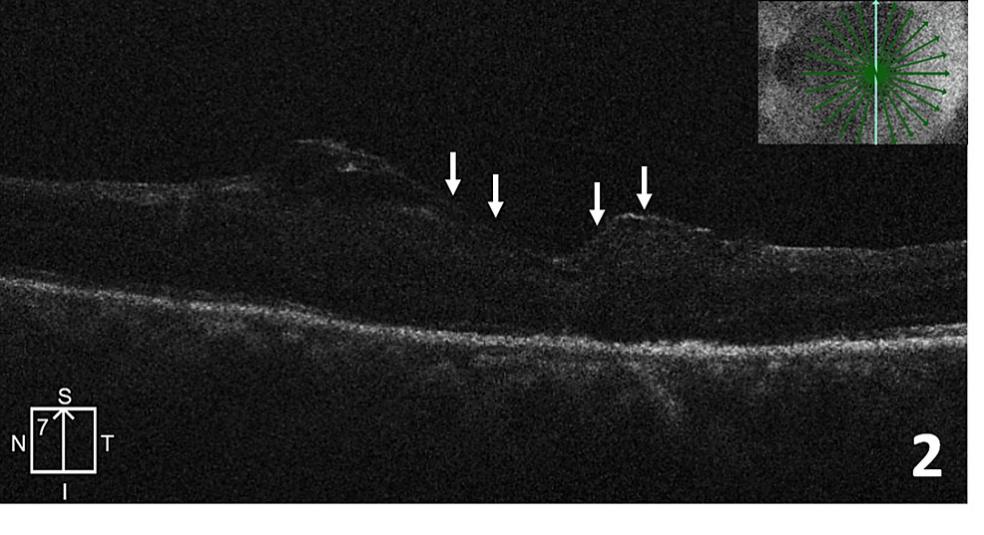
Optical coherence tomography 10 days post capsular flap surgery showing closure of the macular hole with an intact capsular flap covering the fovea (white arrows).

Two months after the initial surgery, he underwent left eye phacoemulsification with posterior chamber intraocular lens (IOL) implantation with revision surgery to address the issue of persistent FTMH. Fluid-air exchange was done to achieve a small rim of residual fluid on the posterior pole. The anterior lens capsule was stained on either side with 0.06% trypan blue solution. No attempt was made to decellularize the lens capsule. A fragment of the stained lens capsule, sized slightly larger than the macular hole was taken into the vitreous cavity using an ILM forceps. It was then unfurled on the fluid over the macular hole and gently stuffed superficially into the edges of the hole. No specific side of the flap was chosen to be placed against the retinal pigment epithelium (RPE). Fluid-air exchange was completed in a controlled manner to maintain the flap in position. Intravitreal 20% SF6 gas tamponade was used at the end of the surgery. The patient was advised to maintain strict prone positioning for one week. Ten days post capsular flap surgery, OCT showed closure of the macular hole with an intact capsular flap covering the fovea and discontinuous outer retinal layers (Figure 2).

**Figure 3:**
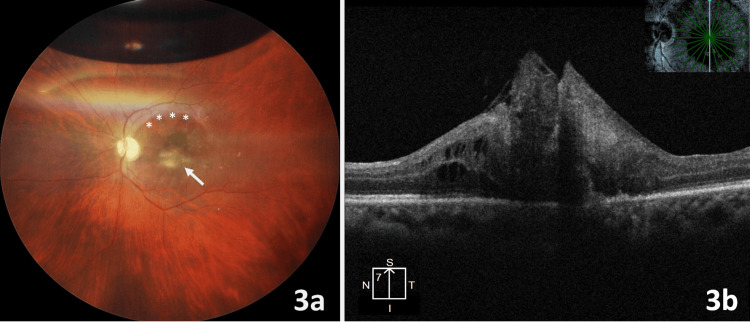
Severe gliosis over the closed hole was noted at three weeks following lens capsular surgery (white arrow). Some fine gliosis was also noted along the superior arcade, marked with white asterisks. A small gas bubble (<1/4th of vitreous volume) is noted in the superior part of the vitreous cavity (A). Optical coherence tomography showing thick proliferation over a closed macular hole with disorganization of foveal layers (B).

At three weeks following lens capsular surgery, severe gliosis was noted over the fovea. Some fine gliosis was also noted along the superior arcade (Figure 3). OCT showed a thick proliferation over a closed macular hole with disorganization of foveal layers. The patient denied any further intervention. Macular gliosis persisted during subsequent follow-ups with vision maintained at 20/200.

## Discussion

With the current standard practice of PPV and ILM peeling with or without flap, macular hole closure is achieved in >90% of cases [6]. Failure of the macular hole to close after primary surgery is often seen in chronic holes, those with larger diameters, high myopia, and non-idiopathic macular holes [6,7]. Our patient had a large FTMH with a minimum linear diameter of 870 µm and also features of chronicity on OCT, like pigmentary changes at the base of the hole, lesser MHI, and rounded edges. These features could have contributed to the failure of closure despite successful ILM peeling with an inverted flap.

Management of persistent macular holes is a challenge. Several techniques have been advocated, ranging from raising flaps of residual ILM, autologous or allogenic material to fill or cover the hole, and manipulation of the macular retina [4-6]. In conventional PPV with ILM peeling, gas prevents fluid entry into the macular hole, aids cell migration, and closes the hole. When an ILM flap is used, it bridges the hole, and gas acts as a stabilizer. ILM peeling can itself provoke gliosis both inside the retina and on the surface of the ILM. The ILM can also act as a scaffold for tissue proliferation [12]. Similarly, a lens capsular flap, like a basement membrane, facilitates the bridging of retinal tissue above the hole [7,11]. Vogt et al. found a positive immunoreactivity of macroglia and microglia cells in a posterior lens capsular flap used to patch a refractory FTMH. Since immunostaining findings of the implanted posterior lens capsular flap and the internal limiting membrane obtained during primary macular hole surgeries are the same, they suggested that lens capsule transplantation can be used as an alternative treatment option for refractory FTMH [13].

Since the description of its use by Chen and Yang, the utility of anterior or posterior lens capsules as a flap to cover persistent macular holes has been well described in the literature [7-11]. The use of a capsular flap has several advantages. It is readily available in most cases either from the anterior lens capsule during cataract surgery in phakic patients or from the posterior lens capsule in pseudophakics [7,8,11]. Since it has a higher density, it settles down over the retinal surface unlike a free ILM flap [7,8]. Being transparent, the lens flap probably does not interfere with visual recovery and helps to achieve good functional as well as anatomical success (closure rates of 96%) [8,10].

Uncontrolled gliosis after ILM peeling is a rare occurrence [14-17]. The incidence of gliosis was reported to be higher in cases where a multi-layered ILM flap was used, high myopes, and where ILM was stuffed into the hole than just inversion of the flap [14-17]. Most studies employing lens capsular flaps for the closure of persistent macular holes have not reported any major capsule-related complications [7,8,11]. The exact cause for such severe gliosis in our case is not known. We propose a few possible mechanisms; the anterior lens capsular flap used in our case was not decellularized, and no specific side was selected to be placed facing the retinal surface. This would have led to the proliferation of the residual lens epithelial cells on the retinal surface causing severe gliosis. Also, a part of the capsule was inserted into the macular hole rather than placed over it, which would have caused microdamage to the retina and RPE inciting a glial response. The role of an exaggerated inflammatory response to a foreign tissue (lens capsule) placed over the retina can also be not ruled out.

Placing a capsular flap with the smoother non-epithelial side would be a better way to prevent the possibility of lens epithelial proliferation. However, the identification of sides in a thin flap, which is manipulated extensively till the time it is placed on the retina, is a challenge. Decellularization of lens epithelium using sterilized distilled water has been described [8,18]. This may be a safer technique to devitalize the capsular epithelium before its placement on the retina and prevent chances of possible epithelial proliferation over the retinal surface.

## Conclusions

Use of lens capsule is an effective technique in the management of persistent macular holes. Some gliosis can occur when macular hole is closed with any material including ILM. Severe gliosis was noted in our case. This could be due to the proliferation of residual lens epithelial cells in the lens capsule, due to microdamage to the retina, or due to an exaggerated inflammatory response to a foreign tissue placed over the retinal surface. Decellularization of lens epithelium may be employed to prevent chances of possible epithelial proliferation over the retinal surface.
